# Difference in the risk of gastrointestinal manifestations between peritoneal and hemodialysis patients: a systematic review and meta-analysis

**DOI:** 10.7717/peerj.21090

**Published:** 2026-04-14

**Authors:** Jiaoyan Li, Jie Liang, Xiaoshuang Shi, Wenjie Lu

**Affiliations:** 1 Nephrology Department, Beijing University of Chinese Medicine Third Affiliated Hospital, Beijing, China; 2 Spleen and Stomach Diseases Department, Beijing University of Chinese Medicine Third Affiliated Hospital, Beijing, China; 3 Beijing University of Chinese Medicine, Beijing, China

**Keywords:** End-stage renal disease, Renal replacement therapy, Gastroesophageal reflux, Digestive, Abdominal

## Abstract

**Objective:**

A large number of patients on dialysis have gastrointestinal (GI) manifestations. However, it remains unclear whether dialysis modality affects their prevalence. We present the first systematic review and meta-analysis of literature comparing the risk of GI manifestations between hemodialysis (HD) and peritoneal dialysis (PD).

**Methods:**

Comparative studies were searched on Embase, PubMed, and Scopus from January 1, 2000, to April 1, 2025. Any GI manifestation reported by at least two studies were quantitatively analysed to obtain the odds ratio (OR) in a random-effect meta-analysis model.

**Results:**

A total of 12 studies were included. Pooled analysis of four studies showed no statistically significant difference in the presence of any GI manifestation between HD and PD. Moreover, the meta-analysis showed no significant differences between the two groups for abdominal pain, anorexia, constipation, diarrhoea, duodenal ulcer, dyspepsia, dysphagia, epigastric pain, esophagitis, gastritis, heartburn, inflammatory bowel disease, nausea, vomiting, pancreatitis, and GI polyps. The risk of cirrhosis, pancreatitis, GI bleeding, and gastric ulcer was significantly higher in HD while the risk of gastroesophageal reflux disease was significantly increased in patients on PD.

**Conclusions:**

Present evidence from a small number of observational studies suggests that majority of GI signs or symptoms do not differ between HD and patients receiving PD. The risk of cirrhosis, pancreatitis, GI bleeding, and gastric ulcer appears higher in HD, whereas the likelihood of gastroesophageal reflux disease is elevated in PD. Observed differences between HD and PD should be interpreted cautiously, as they may partly reflect differences in patient selection, comorbidity profiles, and care pathways rather than a direct causal effect of dialysis modality. Further research can strengthen the quality of the evidence.

## Introduction

Chronic kidney disease (CKD) affects approximately 10% of the global population, and progression to end-stage kidney disease (ESKD) remains a major cause of poor quality of life and premature death (GBD Chronic Kidney Disease Collaboration, 2020). Survival in ESKD patients necessitates kidney replacement therapy or a kidney transplant and patients are frequently offered one of two common dialysis options: peritoneal dialysis (PD) or hemodialysis (HD) (O’connor & Corcoran, 2012). The decision may be influenced by a variety of criteria, including age, comorbidities, cognitive function, lifestyle, associated costs, personal preferences, facility access, and physician recommendation(Torreggiani et al., 2023). Indeed, there exist many differences between HD and PD. In general, patients with PD have greater autonomy and flexibility in dialysis and don’t require repeated hospital visits (Ethier et al., 2024). Additionally, they have better access-site cosmesis and endure less pain from recurrent HD-related cannulations(Shrestha, 2018). It is also recognised that PD has superior early survival rates than HD, with comparable results on extended follow-up (Marshall, 2020). Recent evidence also indicates that patients receiving PD may have a better quality of life, particularly with respect to physical functioning, emotional problems, and sleep quality (Malekmakan et al., 2018; Chuasuwan et al., 2020).

Although patients with CKD and ESKD often suffer from comorbidities such as diabetes and coronary artery disease, the most common, non-kidney, chronic disorders in patients with ESKD are gastrointestinal (GI) disorders. As many as 77%–79% of ESKD patients have GI symptoms (Khan et al., 2025). These symptoms are caused by a wide range of GI illnesses that affect the entire GI system, which occur due to electrolyte imbalance, fluid imbalance, toxin buildup, uremia, medications, dietary and lifestyle constraints, and are consequent to dialysis (Kiziltas, Sahin & Sahin, 2018). According to research, patients with ESKD appear to have a higher prevalence of pancreatitis, ischemic colitis, acute and chronic episodes of GI bleeding, and upper GI lesions than the general population (Kiziltas, Sahin & Sahin, 2018; Khan et al., 2025). Moreover, symptoms like constipation, diarrhoea, bloating, abdominal pain, dyspepsia, nausea, vomiting, and gastroesophageal reflux disease (GERD) can lower the quality of life and affect the physical and mental well-being of patients. However, it is unclear whether the prevalence of GI manifestations varies according to dialysis modality (Zuvela et al., 2018). While numerous studies (Lee et al., 2000; Schoonjans et al., 2002; Yasuda et al., 2002; Kahvecioglu et al., 2005) have compared the GI symptom burden in HD and PD, no meta-analysis has been performed to date. Understanding the symptom burden in HD and among patients receiving PD may help healthcare providers identify modifiable factors to improve symptom treatment and prevention. Therefore, this study aimed to pool data from the literature and compare the risk of various GI manifestations between HD and PD.

## Material and Methods

The protocol for this systematic review and meta-analysis was developed in collaboration with all reviewers and was registered on PROSPERO under the number CRD420251015843 on 20 March 2025. There were no protocol deviations. The review is presented in accordance with PRISMA guidelines (Page et al., 2021).

We used the databases Embase, PubMed, and Scopus to look for articles that compared GI symptoms in patients who received PD and HD. The search was limited to January 1, 2000, through April 1, 2025, to capture only recent data. Two reviewers (JL & XS) searched independently using a mix of MeSH and free keywords which were: “gastric, gastrointestinal, intestinal, digestive, abdominal, peptic, bowel, colon, colorectal, duodenal, constipation, GERD, dysphagia, dyspepsia, indigestion, cirrhosis, fatty liver, gastritis, esophagitis, epigastric pain, diarrhoea, vomiting, anorexia, inflammatory bowel disease (IBD), peritoneal, hemodialysis, end-stage kidney disease”. Search queries used across the databases are shown in Table S1. Two reviewers conducted the search (JL & XS) and differences were handled by discussion with a third reviewer (WL).

After importing the searched articles from the database into EndNote version X9 (Thomson Reuters, New York, NY, USA), duplicate studies were removed. Subsequently, the same two reviewers independently screened the studies for inclusion in the review. This was done by a detailed analysis of the titles and abstracts of the articles. Relevant studies found by either reviewer underwent thorough text analysis before being included. Any differences between the two reviewers (JL & XS) were handled by discussion with a third reviewer (WL). The bibliographies of the included studies and prior reviews were also thoroughly searched for additional relevant articles.

The following eligibility criteria were used to include studies: (1) All study designs with the study population of ESKD under dialysis. (2) Dialysis patients were segregated into HD and PD. (3) The studies compared GI manifestations between PD and HD and reported the number of events of each sign and symptom for each group.

Exclusion criteria were: (1) Studies comparing dialysis patients with controls. (2) Studies reporting only the severity of GI symptoms. (3) Articles using the same database with overlapping study periods. In such cases, the study with the largest sample size was chosen. (4) Non-English language studies and abstracts.

All comparative study designs were considered eligible in order to maximise available evidence, as studies directly comparing GI outcomes between hemodialysis and peritoneal dialysis are limited. To account for methodological variability, a random-effects model was applied for all analyses.

Two reviewers (JL & WL) conducted the data extraction independently. Data were obtained from the studies on the author, publication year, study design, inclusion criteria, sample size, age and gender, method of assessing GI manifestations, and outcome data. We did not pre-determine the GI manifestations to be included, and all outcomes reported by the studies were extracted. The data were cross-checked for errors, and when data were missing for a particular sign or symptom, the study was excluded from the meta-analysis. No data assumptions were made.

The same two investigators (XS & WL) applied the Newcastle Ottawa Scale (NOS) (Wells et al., 2020) to examine study quality and award a quality score of 0–9 to each article. The assessment was done on three domains, which were selection of study participants, comparability of groups by adjustment for confounding, and ascertainment of the exposure or outcome of interest. The total points that can be given for the three domains are four, two, and three, respectively. A higher score indicated better study quality. For the comparability domain of the Newcastle–Ottawa Scale, studies were awarded one point if hemodialysis and peritoneal dialysis groups were matched or adjusted for age and sex, and an additional point if adjustment for other key confounders such as diabetes mellitus or major comorbidities was performed. Studies without matching or multivariable adjustment did not receive points in this domain. Disagreements were resolved in consultation with the third author.

Outcome data was extracted in a Microsoft Excel sheet. Outcomes were broadly categorized as GI manifestations and further classified into: (1) GI symptoms (*e.g.*, abdominal pain, constipation, nausea, vomiting), (2) GI diseases (*e.g.*, cirrhosis, pancreatitis, IBD), and (3) endoscopic or structural findings (*e.g.*, gastric ulcer, GI polyps). If a minimum of two studies were available for an outcome, the data were pooled in “Comprehensive Meta-analysis” (Version 3) software to calculate Odds Ratio (OR) and 95% confidence intervals (CI). All analysis was conducted using a random-effects meta-analysis model using crude 2 *times* 2 data. For outcomes with only one study each, no further analysis was conducted. Given the limited number of studies, we did not assess publication bias. Heterogeneity among studies was assessed through Cochran’s Q statistic and the *I*^2^ index. *I*^2^ of over 50% and/or *P* < 0.05 indicated significant heterogeneity. Subgroup or sensitivity analyses were not performed due to the limited number of studies available for each outcome, as further stratification would have resulted in insufficient data for meaningful comparisons.

## Results

### Search results

**Figure 1:**
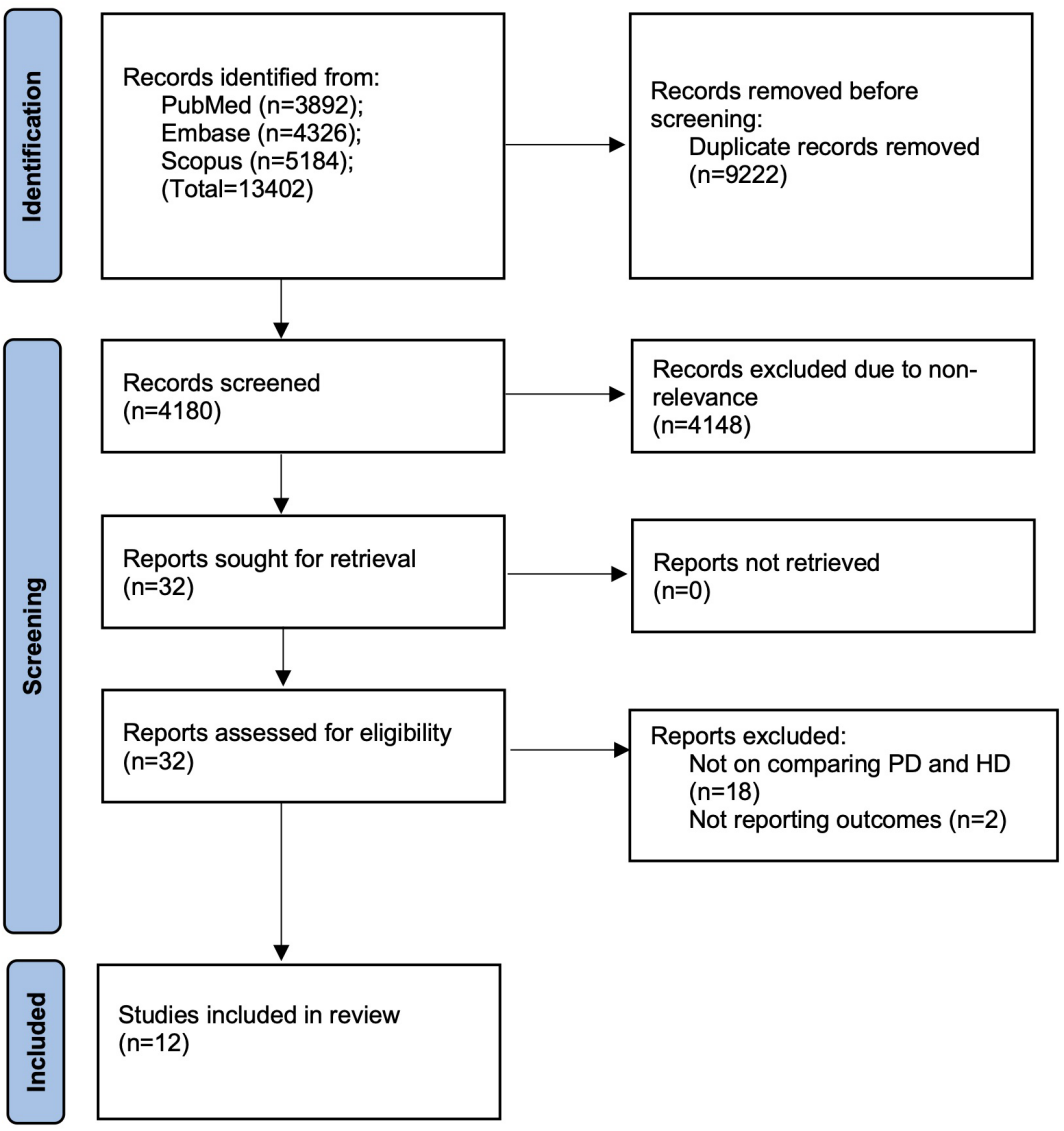
Study flowchart.

The screening and selection process of the studies is illustrated in the PRISMA flowchart of the study (Fig. 1). Following the retrieval of 13,402 papers from the databases, duplicates were removed, and the remaining 4,180 studies were subjected to a thorough screening by the two reviewers. Thirty-two studies were chosen for additional evaluation, with 12 (Lee et al., 2000; Lee et al., 2015; Schoonjans et al., 2002; Yasuda et al., 2002; Kahvecioglu et al., 2005; Cano et al., 2007; Salamon et al., 2013; Song et al., 2013; Zhang et al., 2013; Dong et al., 2014; Usta et al., 2020; Karahan & Şahin, 2022) meeting the inclusion criteria. The inter-rater agreement among the reviewers for study selection was 0.96.

### Baseline details

Details of included studies can be found in Table 1. Published between the years 2000 and 2022, the studies were conducted in either Turkey, China, Korea, Japan, the UK, Australia and Belgium. The largest study was by Lee et al. (2015), which was retrospective cohort study that compared 8,955 patients receiving HD with 1,791 patients receiving PD. All other studies were cross-sectional, with small sample size, ranging from 22 to 478 for HD group and from 19 to 204 for the PD group. All studies included adult dialysis patients with a mean age > 45 years. Several studies also specified the minimum dialysis period for inclusion which ranged from three months to one year. Five studies assessed GI symptoms using patient questionnaires, of which one used the Gastrointestinal Symptom Rating Scale while another used Rome II questionnaire. In the remaining studies, non-standardised questionnaires were used. One study obtained data only from endoscopic findings. In the remaining studies, medical records, patient interviews, physical examination and endoscopic findings were used to record GI manifestations.

**Table 1:** Details of included studies.

Study	Location	Design	Inclusion criteria	Groups	Sample size	Males	Age (years)	Duration of dialysis (months)	Assessment of GI signs/symptoms	NOS score
Karahan & Şahin (2022)	Turkey	CS	>18 years, without acute renal failure, without communication or orientation problems	HD PD	101 92	NR	51.7 ± 15.7 45.4 ± 13.2	23.8 ± 42.4 17.9 ± 28.9	Patient interviews for GI symptoms in the past 6 months, physical examination, prior GI imaging or endoscopy.	Selection: 4 Comparability: 1 Outcome: 1
Usta et al. (2020)	Turkey	CS	>18 years, waiting for kidney transplant	HD PD	22 31	14 20	65.4 ± 11.5 56.2 ± 13.2	46.0 ± 39.6 42.0 ± 45.9	Medical records, endoscopy findings	Selection: 4 Comparability: 0 Outcome: 2
Lee et al. (2015)	Korea	RC	>40 years, without pre-existing GI disease, >3 months on dialysis	HD PD	8955 1791	4673 862	58.6 ± 11.3 55.1 ± 10.3	NR	Medical records	Selection: 4 Comparability: 1 Outcome: 2
Dong et al. (2014)	China	CS	Adults, >3 months on dialysis. Patients with dementia, severe infectious ill- ness, hepatocholecystopathy, peritonitis in the last three months, unstable blood pressure or glucose levels were excluded.	HD PD	182 112	107 61	58.7 ± 14.4 59.7 ± 14.2	55.5 ± 38.5 48.9 ± 31	Modified GSRS questionnaire	Selection: 4 Comparability: 0 Outcome: 2
Zhang et al. (2013)	China	CS	>18 years, >3 months on dialysis, without cognitive disorders or prior abdominal surgery, not having serious illness	HD PD	478 127	257 69	53 ± 14.2 45.2 ± 13.1	53.4 ± 14.9 49.6 ± 10.4	Medical records	Selection: 4 Comparability: 0 Outcome: 2
Song et al. (2013)	Korea	CS	Dialysis for >1year, no history of abdominal surgery or proton pump inhibitor treatment or eradication of *Helicobacter pylori*	HD PD	38 30	17 17	57 ± 9.9 55 ± 11.6	70.5 ± 50.9 61.1 ± 42.2	Endoscopic findings	Selection: 4 Comparability: 0 Outcome: 2
Salamon et al. (2013)	Australia	CS	NR	HD PD	172 122	113 74	63.1 ± 13.5 60.6 ± 14.4	37[14–64] 24[11–37]	Patient interviews	Selection: 4 Comparability: 0 Outcome: 1
Cano et al. (2007)	UK	CS	NR	HD PD	100 48	51 31	NR	NR	Rome II questionnaire	Selection: 3 Comparability: 0 Outcome: 1
Kahvecioglu et al. (2005)	Turkey	CS	>18 years, >6 months on dialysis, albumin levels >4 g/dL and hemoglobin level >9 g/dL, no dementia, no serious illness, not on NSAIDs	HD PD	93 35	NR	NR	NR	Questionnaire	Selection: 3 Comparability: 0 Outcome: 1
Yasuda et al. (2002)	Japan	CS	>21 years, >6 months on dialysis, no peritonitis in the past 6 months, no serious illness, no history of abdominal surgery	HD PD	268 204	165 128	56.4 ± 11.7 50 ± 13.7	NR	Questionnaire	Selection: 4 Comparability: 0 Outcome: 1
Schoonjans et al. (2002)	Belgium	CS	NA	HD PD	66 28	NA	NA	NA	Questionnaire	Selection: 4 Comparability: 0 Outcome: 1
Lee et al. (2000)	Korea	CS	>3 months on dialysis, no major comorbidities	HD PD	22 19	NR	48.8 ± 14.8 47 ± 13.3	NR	Patient interviews	Selection: 4 Comparability: 0 Outcome: 1

**Notes.**

HD hemodialysis PD peritoneal dialysis GI gastrointestinal NOS Newcastle Ottawa scale NR not reported GSRS gastro-intestinal symptom rating scale NA not available CS cross-sectional RC retrospective cohort

### Risk of bias

The risk of bias analysis of every included study is also shown in Table 1. Except for two studies that matched patients receiving HD and PD on age and sex, none of the studies used propensity score matching to minimise baseline differences between the groups. Therefore, most studies did not receive scores for the comparability of groups. Overall, the NOS scores of the studies ranged from four to seven.

### Meta-analysis

We analysed a total of 20 GI manifestations among patients receiving HD and PD. A separate analysis was also conducted for any GI manifestations. Pooled analysis of four studies showed that there was no statistically significant difference in the presence of any GI manifestations between HD and PD. Results of the meta-analyses are summarised in Table 2, while the forest plots are presented as Figs. 2–6 and Figs. S1 to S16.

**Table 2:** Meta-analysis results.

Outcome	Studies	Odds ratio (95% Confidence intervals)	*I* ^2^	Figure number
Any GI manifestation	4	0.83 (0.33, 2.13)	93	Fig. S1
*GI symptoms*				
Abdominal pain	3	1.96 (0.49, 7.79)	90.6	Fig. S2
Anorexia	2	0.48 (0.18, 1.25)	84.2	Fig. S3
Constipation	6	2.26 (0.89, 5.75)	94	Fig. S4
Diarrhea	4	1.29 (0.48, 3.43)	79	Fig. S5
Dyspepsia	4	0.84 (0.31, 2.24)	73	Fig. S6
Dysphagia	2	1.96 (0.81, 4.72)	0	Fig. S7
Epigastric pain	3	2.15 (0.65, 7.07)	64	Fig. S8
Heartburn	3	0.60 (0.32, 1.12)	63	Fig. S9
Nausea	4	0.69 (0.33, 1.45)	71	Fig. S10
Vomiting	4	0.72 (0.31, 1.68)	78	Fig. S11
*GI diseases*				
**Cirrhosis**	**2**	**1.89 (1.38, 2.58)**	**0**	Fig. 2
**Pancreatitis**	**2**	**1.53 (1.03, 2.26)**	**0**	Fig. 3
**GERD**	**6**	**0.72 (0.57, 0.89)**	**0**	Fig. 4
IBD	3	0.75 (0.47, 1.20)	0	Fig. S12
*Endoscopic/structural findings*				
**Gastric ulcer**	**4**	**1.83 (1.62, 2.08)**	**0**	Fig. 5
Duodenal ulcer	2	2.95 (0.38, 22.89)	0	Fig. S13
Gastritis	3	0.67 (0.42, 1.06)	0	Fig. S14
Esophagitis	3	1.28 (0.31, 5.32)	57	Fig. S15
**GI bleeding**	**2**	**1.88 (1.57, 2.24)**	**0**	Fig. 6
Polyp	2	2.81 (0.36, 21.99)	0	Fig. S16

**Notes.**

GI gastrointestinal GERD gastroesophageal reflux disease IBD inflammatory bowel disease

Statistically significant values highlighted in bold.

**Figure 2:**
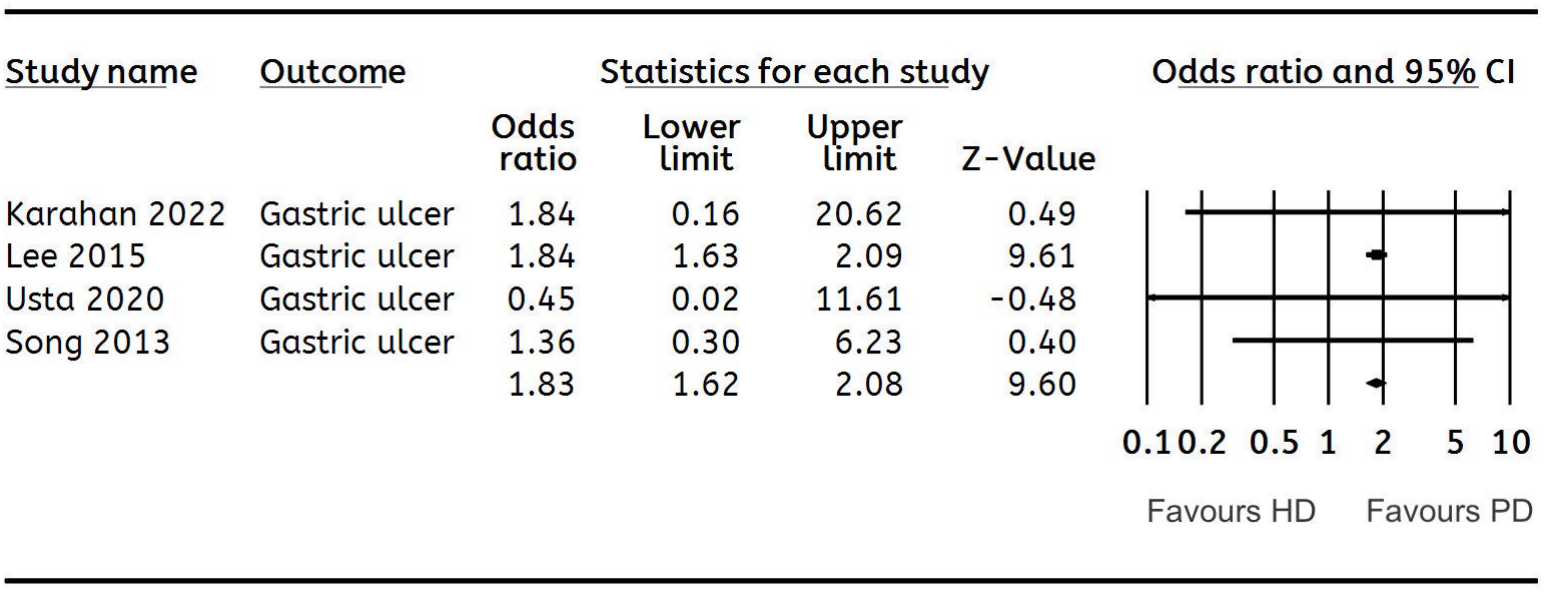
Forest plot showing the meta-analysis of the risk of cirrhosis in HD *versus* PD patients.

**Figure 3:**
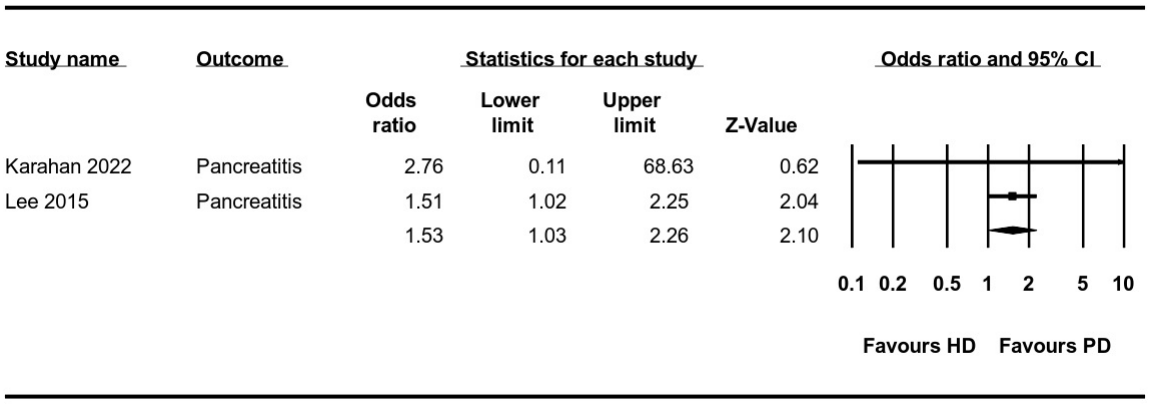
Forest plot showing the meta-analysis of the risk of pancreatitis in HD *versus* PD patients.

**Figure 4:**
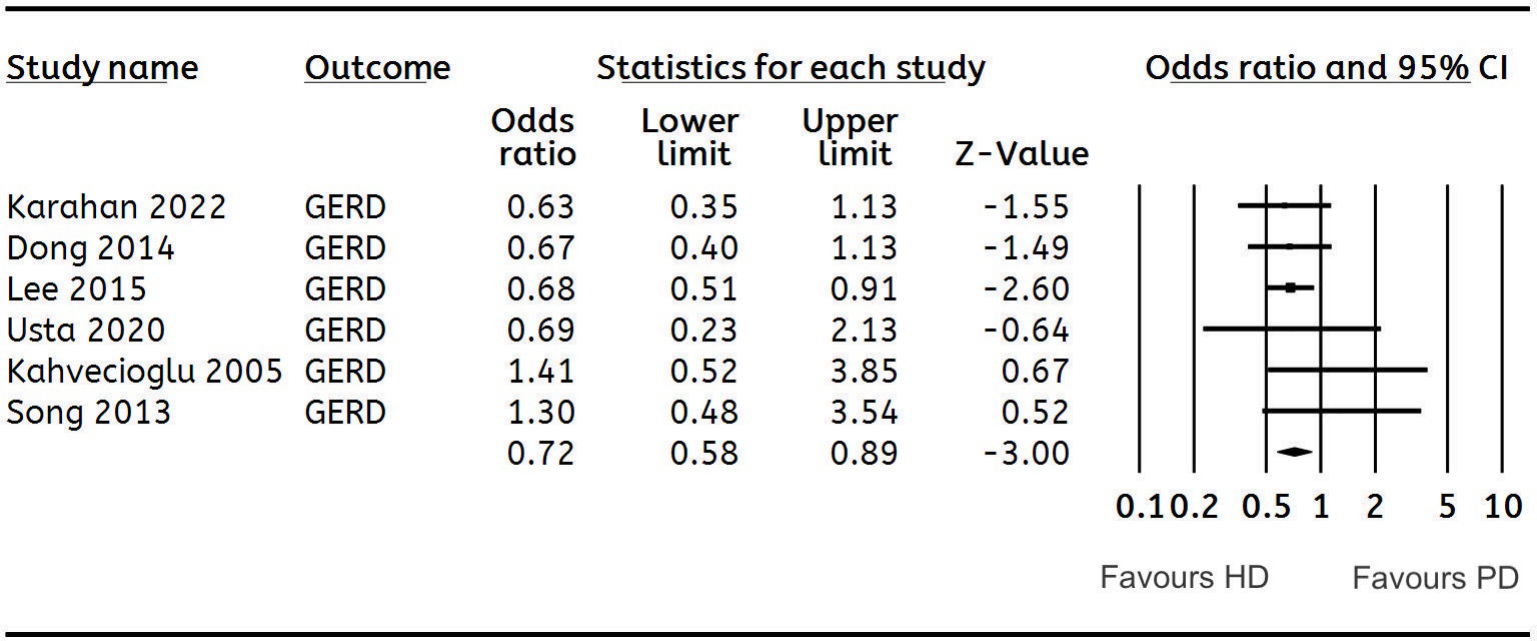
Forest plot showing the meta-analysis of the risk of gastroesophageal reflux disease (GERD) in HD *versus* PD patients.

**Figure 5:**
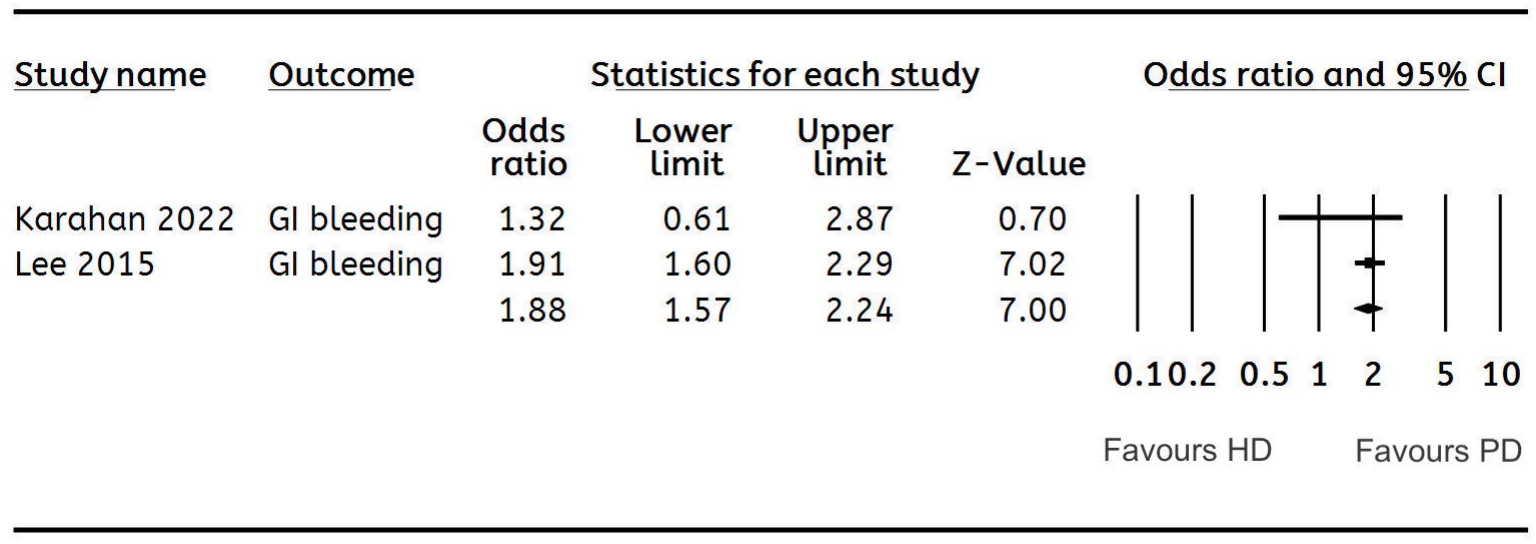
Forest plot showing the meta-analysis of the risk of gastric ulcer in HD *versus* PD patients.

**Figure 6:**
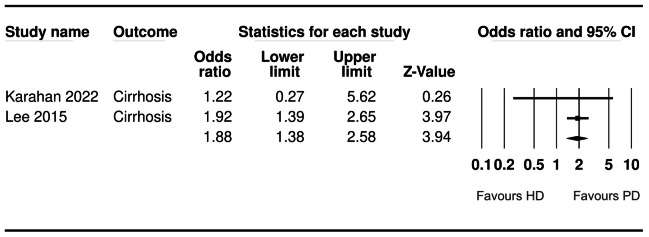
Forest plot showing the meta-analysis of the risk of gastrointestinal bleeding in HD *versus* PD patients.

### GI symptoms

The GI symptoms analysed were abdominal pain, anorexia, constipation, diarrhoea, dyspepsia, dysphagia, epigastric pain, heartburn, nausea, and vomiting. The meta-analysis showed no significant differences between the two groups in any specific GI symptom (Table 2)

### GI diseases

The meta-analysis could pool data on cirrhosis, pancreatitis, IBD, and GERD. Meta-analysis of two studies each showed that the odds of cirrhosis (OR: 1.89 95% CI [1.38–2.58] *I*^2^ = 0%) (Fig. 2) and pancreatitis (OR: 1.53 95% CI [1.03–2.26] *I*^2^ = 0%) (Fig. 3) were significantly higher in HD as compared to PD. The risk of GERD between the two groups was reported by six studies. Pooled analysis showed that patients receiving HD had a significantly lower risk of GERD as compared to those on PD (OR: 0.72 95% CI [0.57–0.89] *I*^2^ = 0%) (Fig. 4). We conducted a sensitivity analysis by excluding the largest study of Lee et al. (2015) for this analysis and the results showed no difference in the risk of GERD between the two groups (OR: 0.77 95% CI [0.55–1.06] *I*^2^ = 0%). However, there was no difference in the risk of IBD (Table 2).

### Endoscopic/structural findings

Data on gastric ulcer, duodenal ulcer, gastritis, esophagitis, GI bleeding and polyps were pooled for a meta-analysis. A meta-analysis of four and two studies showed that patients on HD had a significantly higher risk of gastric ulcers (OR: 1.83 95% CI [1.62–2.08] *I*^2^ = 0%) (Fig. 5) and GI bleeding (OR: 1.88 95% CI [1.57–2.24] *I*^2^ = 0%) (Fig. 6) as compared to those on PD. However, none of the remaining findings were found to be significantly different between the two groups (Table 2). We also conducted a sensitivity analysis by excluding the largest study of Lee et al. (2015) for gastric ulcer. After exclusion of the study, the results turned non-significant indicating no difference in the risk of gastric ulcer with either dialysis modality (OR: 1.26 95% CI [0.38–4.18] *I*^2^ = 0%).

## Discussion

GI manifestations are commonly observed in individuals who have been diagnosed with CKD of any stage. Recent studies indicate that approximately 90% of CKD patients exhibit GI symptoms, with an average of four symptoms per patient (Karahan & Şahin, 2022). Moreover, research shows that GI findings are noted in both CKD and kidney transplant recipients (Thomas et al., 2013). According to their data, diarrhoea was seen in over half of the kidney transplant recipients, with infection and drug-related colitis being the commonest aetiology. Another study shows that uremic patients (CKD, HD) had a higher prevalence of erosive gastritis and duodenal ulcers as compared to transplant recipients and controls (Khedmat et al., 2007). Further variations have also been suggested based on treatment modalities. Salamon et al. (2013) comparing patients receiving PD and HD noted the prevalence of GI symptoms was much higher in PD (85%) as compared to HD (51%). Long-term exposure to acidic dialysate with high glucose and breakdown products alters the peritoneal membrane and may contribute to increased GI manifestations in PD (Perl & Bargman, 2016). Importantly, GI symptoms can negatively affect food intake in dialysis patients, contributing to malnutrition (Salamon et al., 2013). Malnutrition, in turn, has been found to strongly predict mortality in both HD and PD (Rashid et al., 2024). Given the adverse impact of GI symptoms on outcomes in ESKD, it is necessary to examine how GI symptoms differ between HD and PD. As a result, the current study provides important evidence, as it is the first to pool data on the risk of approximately 20 GI manifestations between PD and HD.

At the outset, it must be pointed out that the present data is from a limited number of studies and mostly with a small sample size. At most, only two to six studies could be included in each meta-analysis, and hence, results must be interpreted with caution. We noted that the presence of any GI manifestations did not differ between patients receiving PD and HD. High heterogeneity was observed in the meta-analysis, indicating substantial variability across individual studies. When GI manifestations were examined by outcome category, most patient-reported GI symptoms did not differ significantly between HD and PD. In contrast, differences were more consistently observed for specific GI diseases (cirrhosis, pancreatitis) and endoscopic or structural findings, particularly ulcer- and bleeding-related outcomes, which were more frequent among patients receiving HD, and GERD, which was more common in patients receiving PD. All of these outcomes had low inter-study heterogeneity, providing some confidence in the results. However, the results of gastric ulcer and GERD seemed to be influence by the large study of Lee et al. (2015), as the results turned non-significant on sensitivity analysis. Importantly, uremic symptoms like nausea, vomiting, and anorexia, which are among the commonest symptoms noted in ESKD (Cano et al., 2007), did not differ between HD and PD. Meta-analysis also showed a higher prevalence of constipation in HD than in PD, but the difference did not reach statistical significance. Research shows that passive behaviour, dehydration, diminished fibre consumption (attributable to potassium-restricted diets), metabolic irregularities, phosphate binders, aluminium antacids, ion-exchange resins, comorbidities, and extended colonic transit time may be linked to the occurrence of constipation in individuals with CKD (Costa-Moreira et al., 2020). Laxative use is especially important in patients on PD and can affect their GI symptom profile. Constipation, a known risk factor, can lead to PD catheter dysfunction and is linked to peritonitis caused by disrupted dialysate flow, higher intra-abdominal pressure, and bacterial translocation. Therefore, routine bowel management, including the prophylactic or regular use of laxatives, is often recommended in PD to maintain catheter patency and prevent infections (Zhang et al., 2013). However, most included studies did not systematically report laxative use, limiting our ability to assess its independent effect on GI outcomes.

Overall, CKD patients have a 10–12-fold higher risk of gastric ulcers compared with the general population (Liang et al., 2014). Population-based studies also show that CKD patients have a higher risk of gastric ulcer bleeding and bleeding-related morbidity and mortality (Luo et al., 2011; Yang et al., 2012). Liang et al. (2014) have shown that HD remains a significant risk factor for gastric ulcers in CKD patients regardless of the duration, increasing the risk by about 10-fold. The higher prevalence of gastric ulcers in HD *vs* PD noted in our meta-analysis can be due to several factors the use of anticoagulation during HD, intradialytic hypotension and hemodynamic changes during the procedure. Intradialytic hypotension is still one of the most common HD issues, affecting around 20–30% of HD sessions. Stress ulcer-like mucosal lesions are likely to develop in such patients, as hypotension causes splanchnic hypoperfusion and subsequently GI mucosal ischemia (Liang et al., 2014; Sars, Van der Sande & Kooman, 2020). The use of gastric irritants like non-steroidal anti-inflammatory drugs is also found to be higher in HD *vs* PD which may contribute to gastric ulcers (Heleniak et al., 2017). The elevated risk of GI bleeding in HD *vs* PD can be consequent to the high prevalence of gastric ulcers. According to a recent meta-analysis, the pooled prevalence of GI bleeding in CKD patients is 2.2%, with rates increasing to 35.8% when endoscopic evaluation is undertaken (Lin et al., 2023). Moreover, the study also noted that HD is an independent predictor of GI bleeding. In contrast to patients receiving PD, the high incidence of gastric ulcers and the use of anticoagulation during HD can be a significant cause of GI bleeding (Lin et al., 2023).

The meta-analysis on cirrhosis and pancreatitis could include just two studies of which one Lee et al. (2015) was with a very large sample size. This study reported a higher risk of cirrhosis and pancreatitis among HD patients, resulting in a positive effect size. However, in their multivariate analysis, the authors noted that after adjustment for age, gender, and comorbidities, there was no difference in the risk of cirrhosis (OR: 0.80 95% CI [0.58–1.10]) and pancreatitis (OR: 0.91 95% CI [0.60–1.36]) between HD and PD.

The meta-analysis also showed a significantly higher risk of GERD in PD as compared to HD. However, this analysis too was affected by the large study of Lee et al. (2015), which found a significantly lower risk of GERD in PD, while all the other studies did not note any such difference. It is suggested that the filling of the abdominal cavity with dialysate fluid during PD can worsen GERD symptoms by lowering the oesophagal sphincter pressure and raising intra-abdominal pressure, leading to a higher frequency of acid reflux episodes. Furthermore, the glucose dialysate may have a metabolic role in gastric emptying, leading to delay and increased risk of GERD in patients receiving PD (Dong et al., 2014). However, there have been opposing results as well. A manometric study found that dialysate infusion did not affect lower oesophagal sphincter pressures, and GERD symptoms may be unrelated to changes in gastric and esophageal pressures (Hylander et al., 1991). Given the limited data, further studies are needed to provide better evidence.

Emerging research indicates that gut dysbiosis plays a significant role in the pathogenesis of GI symptoms in patients with ESKD (Stadlbauer et al., 2017; Luo et al., 2021). Uremia is associated with substantial alterations in the gut microbiota, characterised by reduced bacterial diversity and overgrowth of urease-, indole-, and p-cresol-producing bacteria. These microbial shifts compromise intestinal barrier integrity, elicit localised inflammatory responses, and increase the production of uremic toxins, which may underlie symptoms such as abdominal discomfort, bloating, diarrhoea, constipation, and nausea (Luo et al., 2021). The modality of dialysis may further influence these phenomena. HD has been associated with heightened hemodynamic instability and intestinal hypoperfusion, potentially exacerbating mucosal ischemia and impairing barrier function, thereby intensifying dysbiosis-related inflammation (Du et al., 2024). Conversely, PD may alter gut microbiota through mechanisms such as chronic exposure to glucose-rich dialysate, increased intra-abdominal pressure, and modifications in intestinal transit, which are hypothesized to promote gastroesophageal reflux and microbial composition changes (Stepanova, 2023). Although direct comparative studies examining microbiota profiles between HD and PD are limited, these modality-specific physiological differences offer a plausible biological basis for distinct GI manifestations which needs to be explored by further studies.

This review has several limitations. The small number of studies, with predominantly observational cross-sectional designs and many with limited sample sizes, precludes strong conclusions. Variations in data reporting prevented inclusion of all studies in the meta-analysis. Moreover, only selected and most commonly reported GI manifestations could be analysed based on the availability of data. The diagnosis or identification of outcomes was based on either medical records, patient questionnaires or interviews. Each of these methods has drawbacks. Misclassification of GI symptoms may have led to bias. It is also plausible that several symptoms may have gone unreported due to recall bias or mild severity of the symptom. The short follow-up of studies is another limitation and patients might have not reported early symptoms. A key limitation of this meta-analysis is the limited adjustment for confounding in the included studies. Only a small number of studies accounted for baseline differences such as age, sex, or diabetes mellitus, and most analyses were based on unadjusted comparisons between HD and PD groups. Important confounders, including comorbidity burden, dialysis duration, medication use (such as anticoagulants, non-steroidal anti-inflammatory drugs, and acid-suppressive therapy), nutritional status, and lifestyle factors, were inconsistently reported and could not be incorporated into pooled analyses. Based on this, most included studies received low scores in the comparability domain of the NOS, reflecting the lack of matching or adjustment for baseline characteristics between HD and PD groups. Consequently, the pooled estimates from this meta-analysis should be interpreted with caution. Additionally, formal assessment of publication bias was not feasible due to the limited number of studies per outcome, the possibility of publication bias cannot be excluded. Several included studies had small sample sizes and reported multiple GI outcomes, raising the potential for selective outcome reporting. However, for outcomes demonstrating statistically significant associations, the direction of effect was consistent across contributing studies and inter-study heterogeneity was minimal, suggesting that the findings are unlikely to be driven solely by publication bias. Several outcomes, particularly the presence of any GI symptom, demonstrated substantial heterogeneity. This likely reflects clinical and methodological diversity among included studies, including differences in study design, geographic region, dialysis duration, and methods used to assess GI symptoms (questionnaires, interviews, medical records, or endoscopic findings). Subjective symptom-based outcomes are especially prone to such variability. Formal subgroup or sensitivity analyses were not feasible because only a small number of studies contributed to most outcomes, and further stratification would have resulted in single-study subgroups. Importantly, outcomes showing statistically significant associations exhibited minimal inter-study heterogeneity, lending confidence to these findings. For highly heterogeneous outcomes, pooled estimates should be interpreted with caution.

This study’s strength is that it offers the first meta-analysis comparing GI manifestations between patients receiving HD and PD. The findings have clinical implications: they highlight differences in GI symptoms that can inform patient-doctor discussions on dialysis choices. Patients on HD may need more frequent endoscopies for gastric ulcers and GI bleeding; patients receiving PD should be aware of GERD risks and consult gastroenterologists early. Patients receiving PD often have less access to dietary services as they dialyse at home, unlike patients receiving HD who receive dietary advice at satellite centres. Increased dietary support is needed for patients receiving PD to prevent malnutrition.

## Conclusions

Meta-analysis of a limited number of observation studies suggests that the risk of GI symptoms may not differ between patients receiving HD or PD. The risk of cirrhosis, pancreatitis, GI bleeding, and gastric ulcer appears higher in HD, whereas the likelihood of GERD is elevated in PD. Observed differences between HD and PD should be interpreted cautiously, as they may partly reflect differences in patient selection, comorbidity profiles, and care pathways rather than a direct causal effect of dialysis modality. Further studies can help strengthen the quality of the evidence.

## Supplemental Information

10.7717/peerj.21090/supp-1 Supplemental Information 1 PRISMA checklist

10.7717/peerj.21090/supp-2 Supplemental Information 2 Search queries used to retrieve articles

10.7717/peerj.21090/supp-3 Supplemental Information 3 Meta-analysis of the risk of any GI symptoms between HD and PD

10.7717/peerj.21090/supp-4 Supplemental Information 4 Meta-analysis of the risk of abdominal pain between HD and PD

10.7717/peerj.21090/supp-5 Supplemental Information 5 Meta-analysis of the risk of anorexia between HD and PD

10.7717/peerj.21090/supp-6 Supplemental Information 6 Forest plot showing the meta-analysis of the risk of constipation in HD versus PD patients

10.7717/peerj.21090/supp-7 Supplemental Information 7 Forest plot showing the meta-analysis of the risk of diarrhea in HD versus PD patients

10.7717/peerj.21090/supp-8 Supplemental Information 8 Forest plot showing the meta-analysis of the risk of dyspepsia in HD versus PD patients

10.7717/peerj.21090/supp-9 Supplemental Information 9 Forest plot showing the meta-analysis of the risk of dysphagia in HD versus PD patients

10.7717/peerj.21090/supp-10 Supplemental Information 10 Forest plot showing the meta-analysis of the risk of epigastric pain in HD versus PD patients

10.7717/peerj.21090/supp-11 Supplemental Information 11 Forest plot showing the meta-analysis of the risk of heartburn in HD versus PD patients

10.7717/peerj.21090/supp-12 Supplemental Information 12 Forest plot showing the meta-analysis of the risk of nausea in HD versus PD patients

10.7717/peerj.21090/supp-13 Supplemental Information 13 Forest plot showing the meta-analysis of the risk of vomiting in HD versus PD patients

10.7717/peerj.21090/supp-14 Supplemental Information 14 Forest plot showing the meta-analysis of the risk of inflammatory bowel disease (IBD) in HD versus PD patients

10.7717/peerj.21090/supp-15 Supplemental Information 15 Forest plot showing the meta-analysis of the risk of duodenal ulcer in HD versus PD patients

10.7717/peerj.21090/supp-16 Supplemental Information 16 Forest plot showing the meta-analysis of the risk of gastritis in HD versus PD patients

10.7717/peerj.21090/supp-17 Supplemental Information 17 Forest plot showing the meta-analysis of the risk of esophagitis in HD versus PD patients

10.7717/peerj.21090/supp-18 Supplemental Information 18 Forest plot showing the meta-analysis of the risk of gastrointestinal polyps in HD versus PD patients

10.7717/peerj.21090/supp-19 Supplemental Information 19 Meta analysis dataset

## Additional Information and Declarations

### Data Availability

The following information was supplied regarding data availability: This article is a systematic review/meta-analysis.
