# Supplementary material for: Difference in the risk of gastrointestinal manifestations between peritoneal and hemodialysis patients: a systematic review and meta-analysis
Source: PeerJ. 2026 Apr 14;14:e21090. doi: 10.7717/peerj.21090 (PMC13089217; doi:10.7717/peerj.21090)
Supplement: Supplemental Information 2 [file peerj-14-21090-s002.docx]

Supplementary Table 1: Search queries used to retrieve articles

**PubMed**

(gastric[Title/Abstract] OR gastrointestinal[Title/Abstract] OR intestinal[Title/Abstract]

OR digestive[Title/Abstract] OR abdominal[Title/Abstract] OR peptic[Title/Abstract]

OR bowel[Title/Abstract] OR colon[Title/Abstract] OR colorectal[Title/Abstract]

OR duodenal[Title/Abstract] OR constipation[Title/Abstract]

OR gastroesophageal reflux[Title/Abstract] OR dysphagia[Title/Abstract]

OR dyspepsia[Title/Abstract] OR indigestion[Title/Abstract]

OR cirrhosis[Title/Abstract] OR fatty liver[Title/Abstract]

OR gastritis[Title/Abstract] OR esophagitis[Title/Abstract]

OR epigastric pain[Title/Abstract] OR diarrhea[Title/Abstract]

OR vomiting[Title/Abstract] OR anorexia[Title/Abstract]

OR inflammatory bowel disease[Title/Abstract])

AND

(peritoneal dialysis[MeSH Terms] OR peritoneal dialysis[Title/Abstract])

AND

(hemodialysis[MeSH Terms] OR hemodialysis[Title/Abstract])

**Embase**

('gastrointestinal disease'/exp OR gastric:ti,ab OR gastrointestinal:ti,ab

OR intestinal:ti,ab OR digestive:ti,ab OR abdominal:ti,ab OR peptic:ti,ab

OR bowel:ti,ab OR colon:ti,ab OR colorectal:ti,ab OR duodenal:ti,ab

OR constipation:ti,ab OR 'gastroesophageal reflux':ti,ab

OR dysphagia:ti,ab OR dyspepsia:ti,ab OR indigestion:ti,ab

OR cirrhosis:ti,ab OR 'fatty liver':ti,ab OR gastritis:ti,ab

OR esophagitis:ti,ab OR 'epigastric pain':ti,ab OR diarrhea:ti,ab

OR vomiting:ti,ab OR anorexia:ti,ab OR 'inflammatory bowel disease':ti,ab)

AND

('peritoneal dialysis'/exp OR 'peritoneal dialysis':ti,ab)

AND

('hemodialysis'/exp OR hemodialysis:ti,ab)

**Scopus**

TITLE-ABS-KEY (gastric OR gastrointestinal OR intestinal OR digestive OR abdominal

OR peptic OR bowel OR colon OR colorectal OR duodenal

OR constipation OR "gastroesophageal reflux" OR dysphagia

OR dyspepsia OR indigestion OR cirrhosis OR "fatty liver"

OR gastritis OR esophagitis OR "epigastric pain"

OR diarrhea OR vomiting OR anorexia OR "inflammatory bowel disease")

AND

TITLE-ABS-KEY (peritoneal)

AND

TITLE-ABS-KEY (hemodialysis)
