# Supplementary figures and images for: Difference in the risk of gastrointestinal manifestations between peritoneal and hemodialysis patients: a systematic review and meta-analysis
Source: PeerJ. 2026 Apr 14;14:e21090. doi: 10.7717/peerj.21090 (PMC13089217; doi:10.7717/peerj.21090)

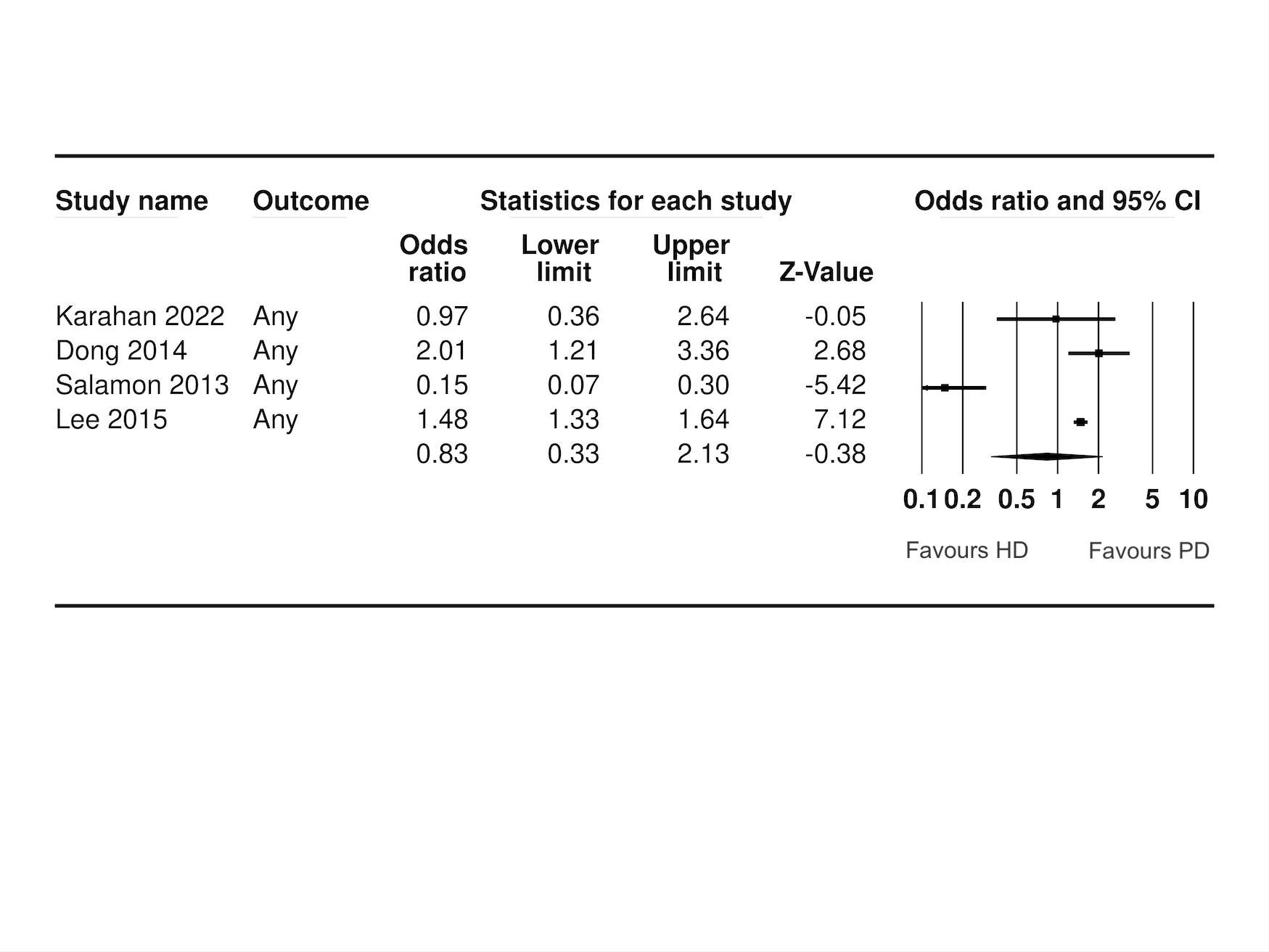

Supplement: Supplemental Information 3 [file peerj-14-21090-s003.tiff]

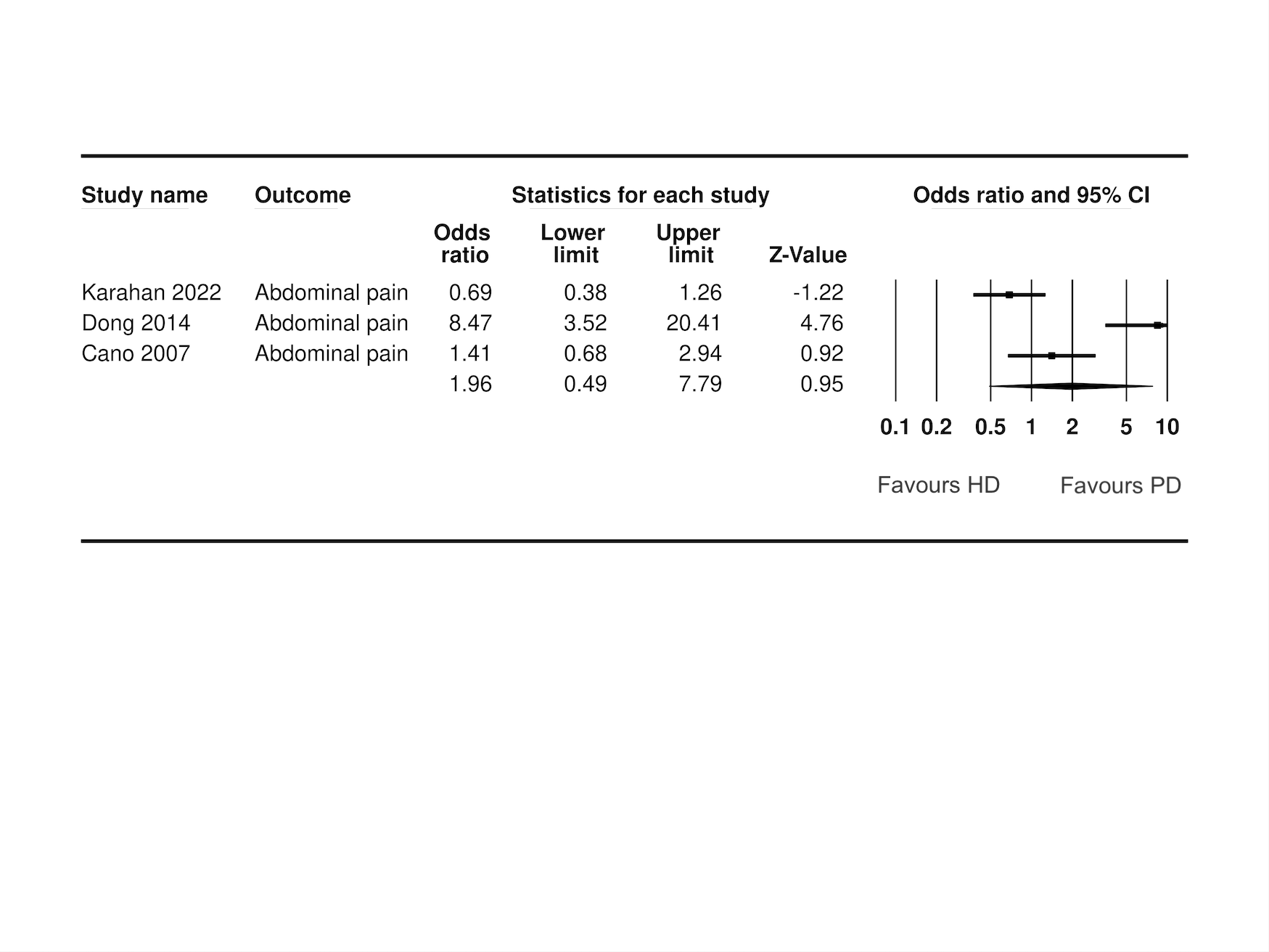

Supplement: Supplemental Information 4 [file peerj-14-21090-s004.tiff]

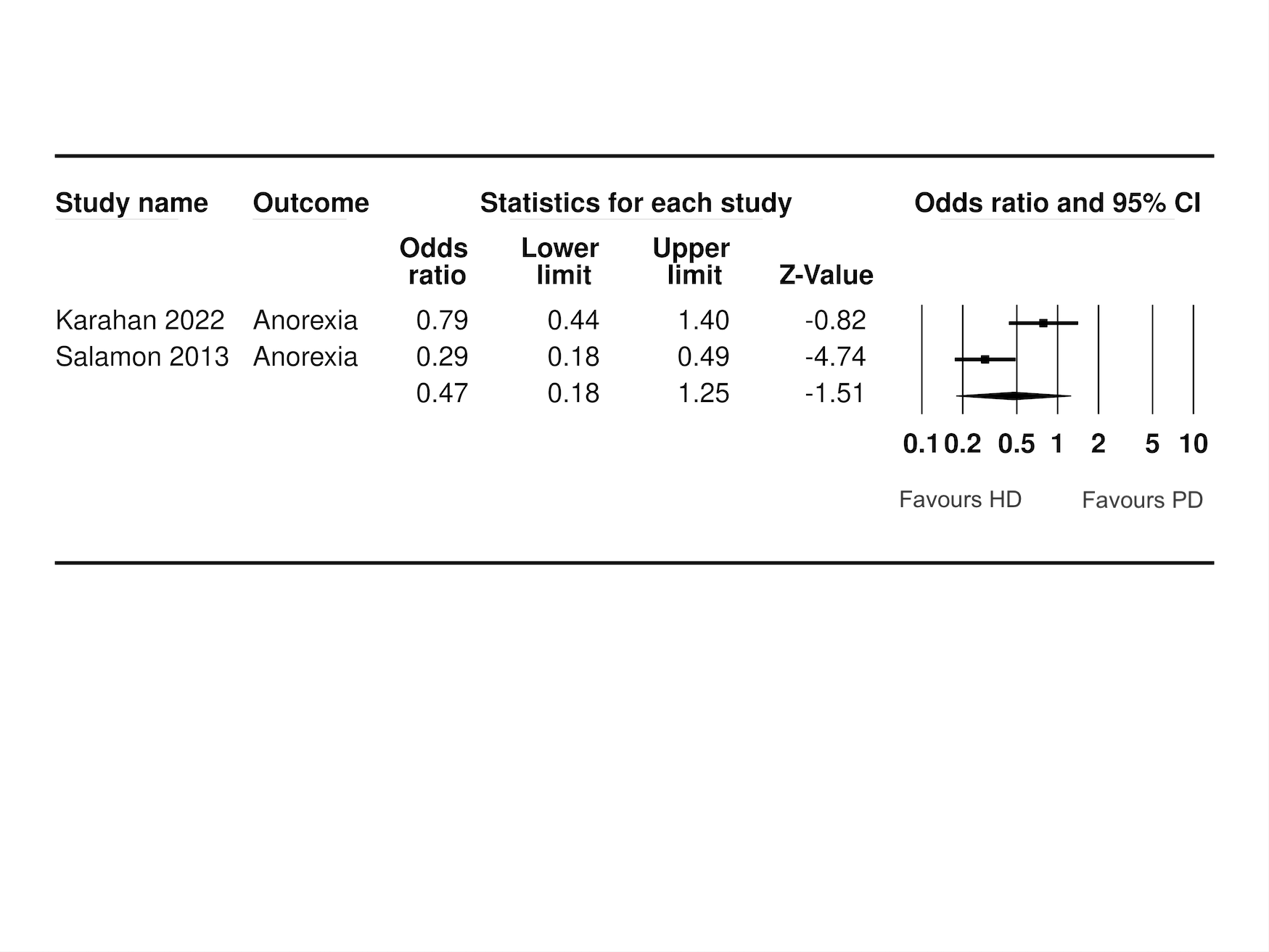

Supplement: Supplemental Information 5 [file peerj-14-21090-s005.tiff]

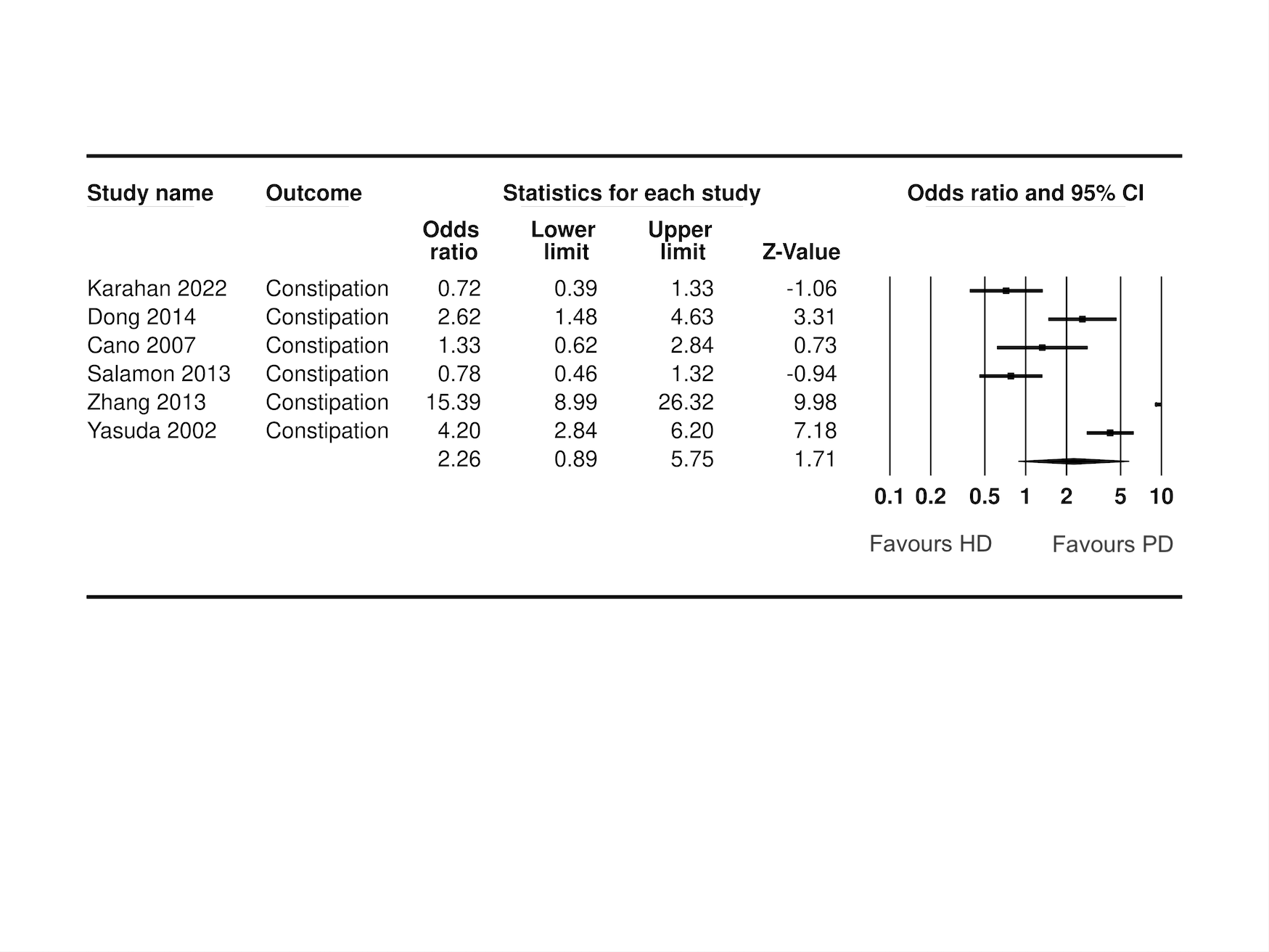

Supplement: Supplemental Information 6 [file peerj-14-21090-s006.tiff]

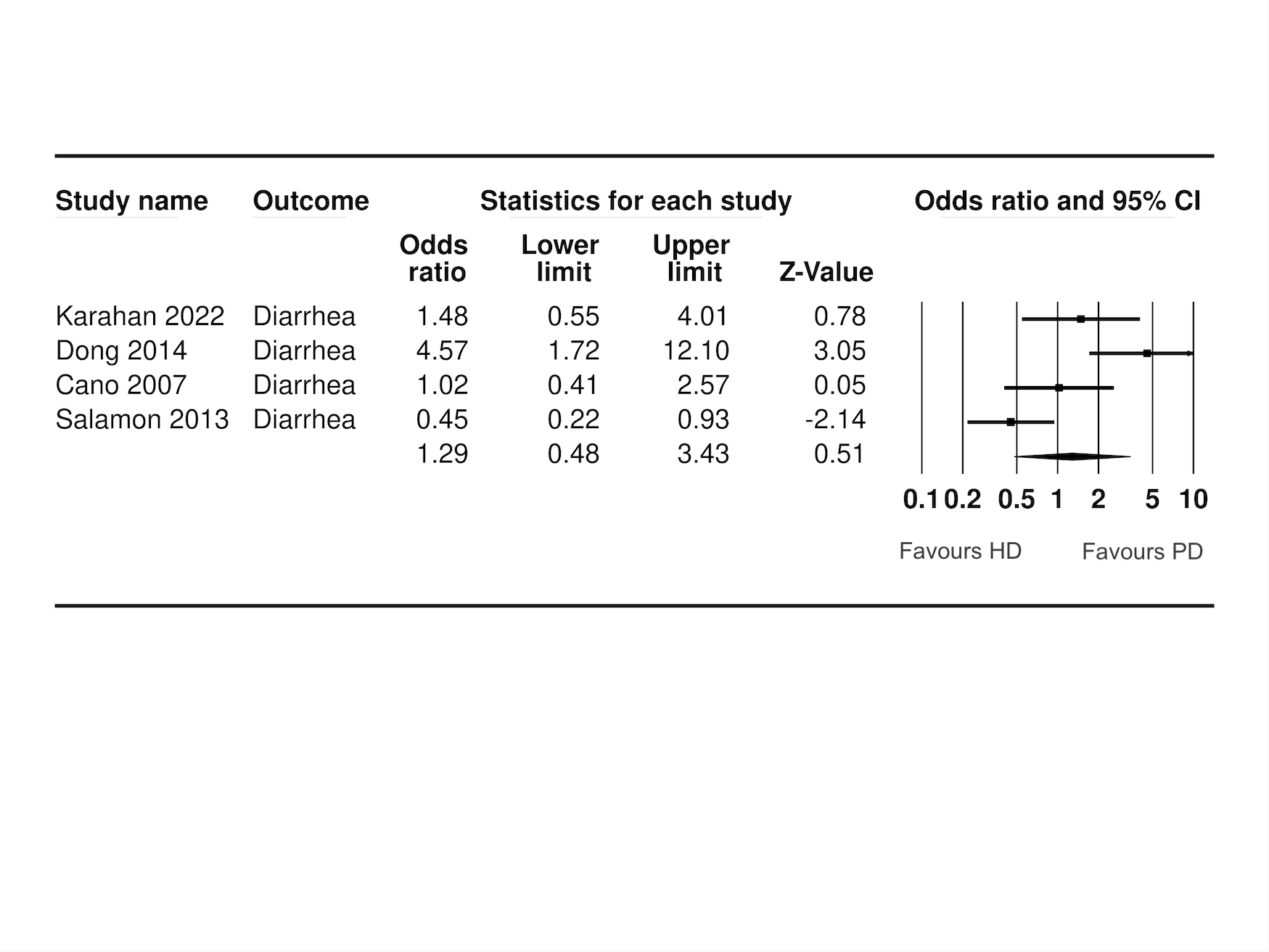

Supplement: Supplemental Information 7 [file peerj-14-21090-s007.tiff]

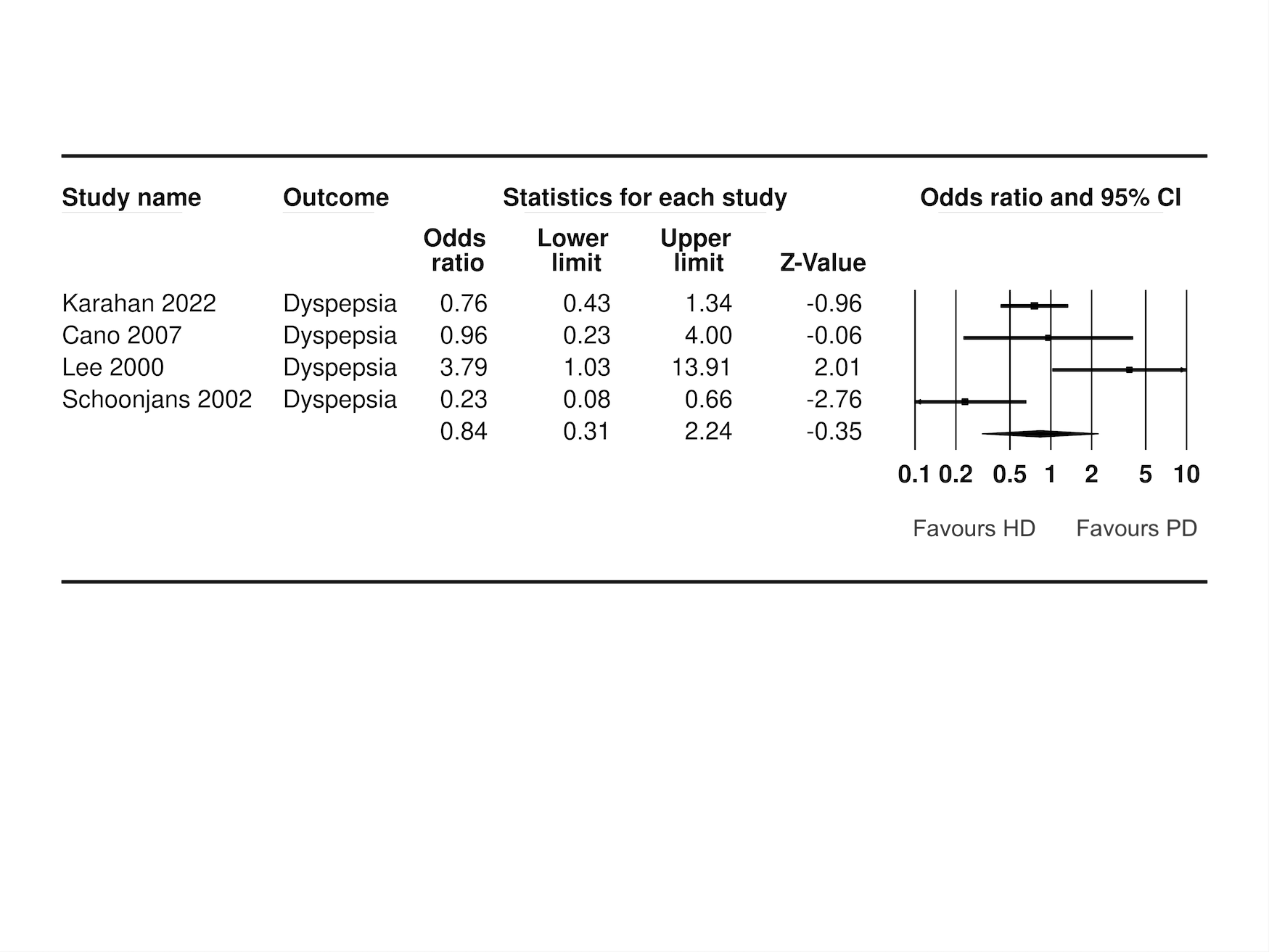

Supplement: Supplemental Information 8 [file peerj-14-21090-s008.tiff]

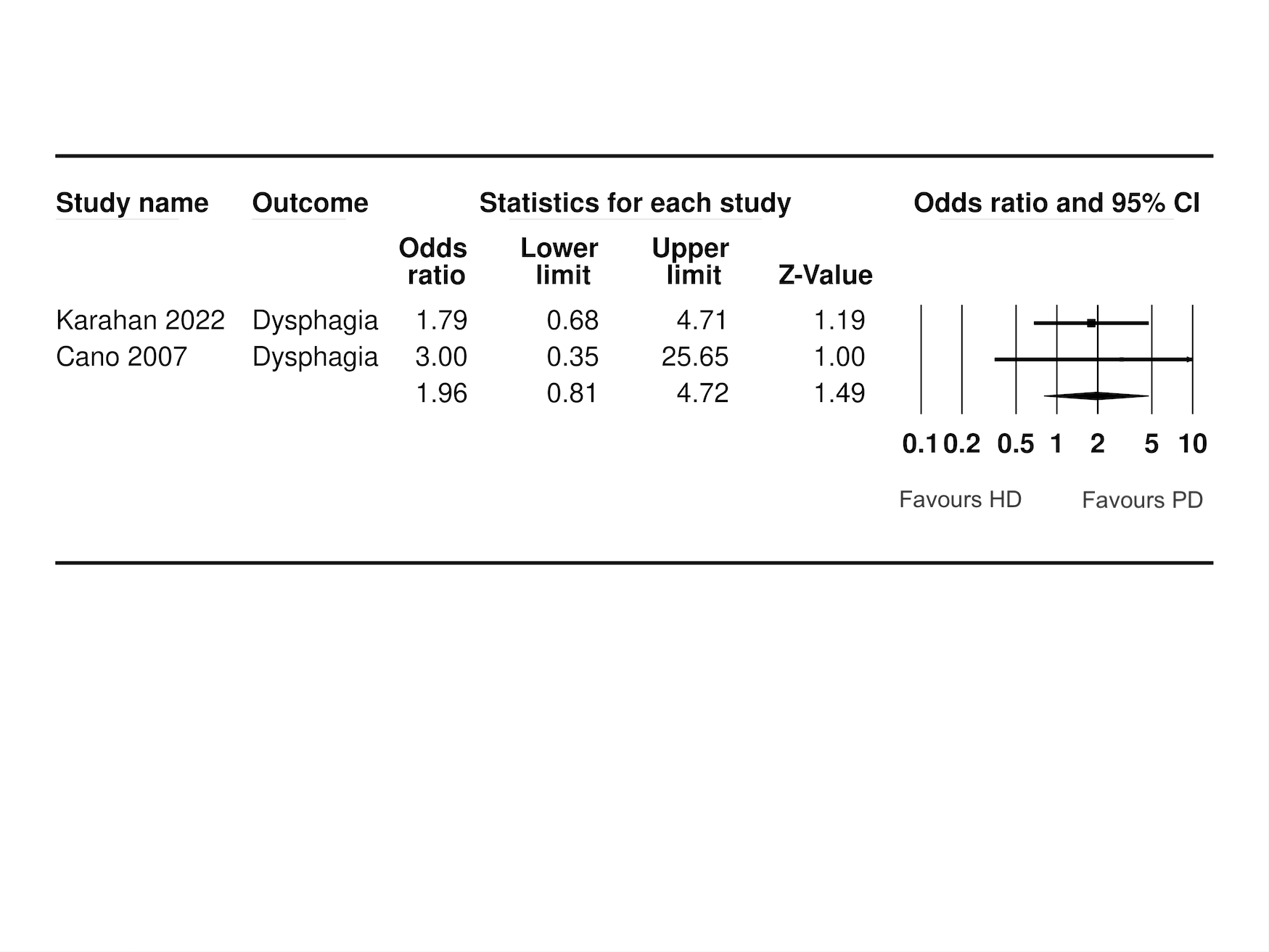

Supplement: Supplemental Information 9 [file peerj-14-21090-s009.tiff]

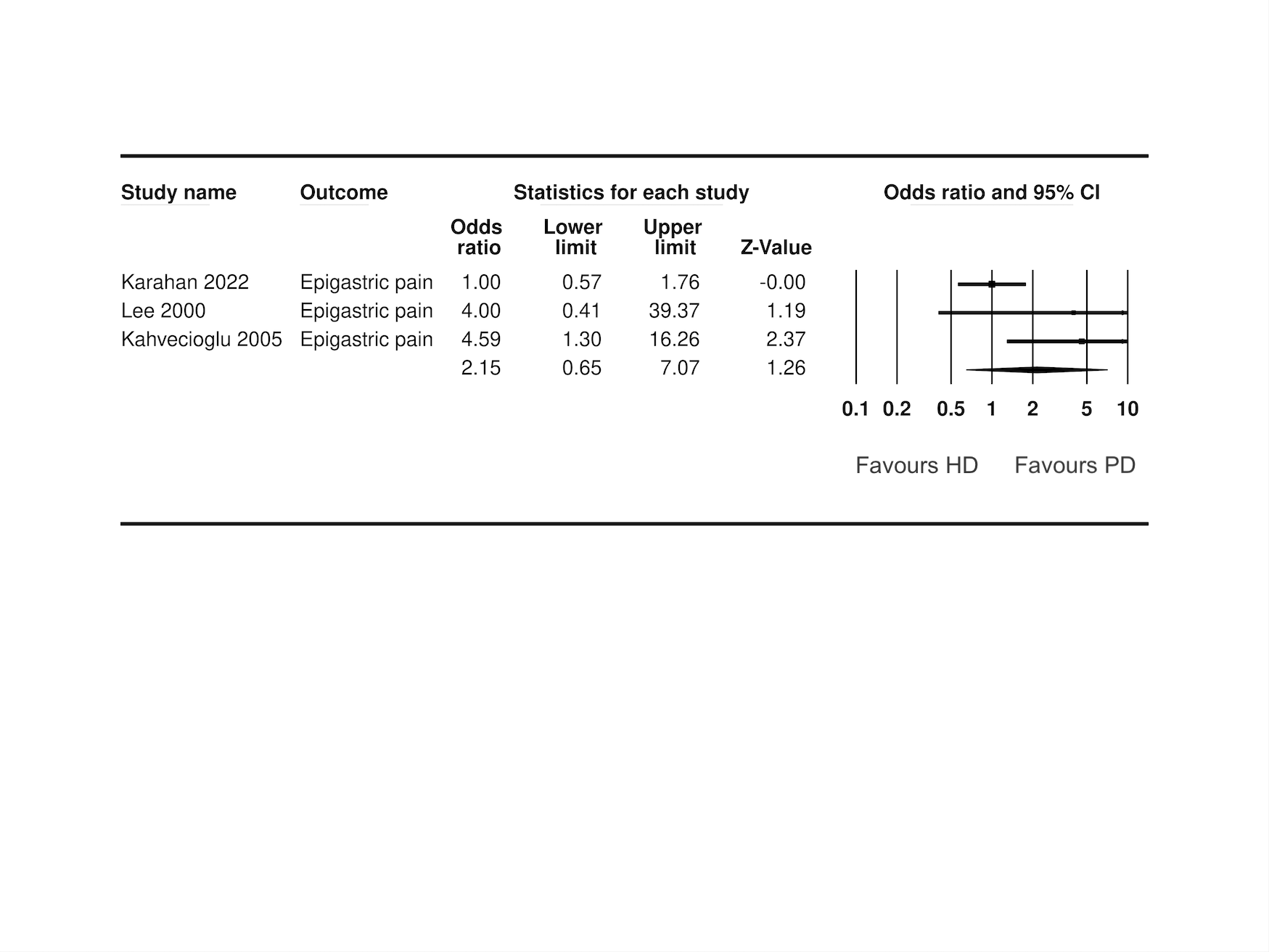

Supplement: Supplemental Information 10 [file peerj-14-21090-s010.tiff]

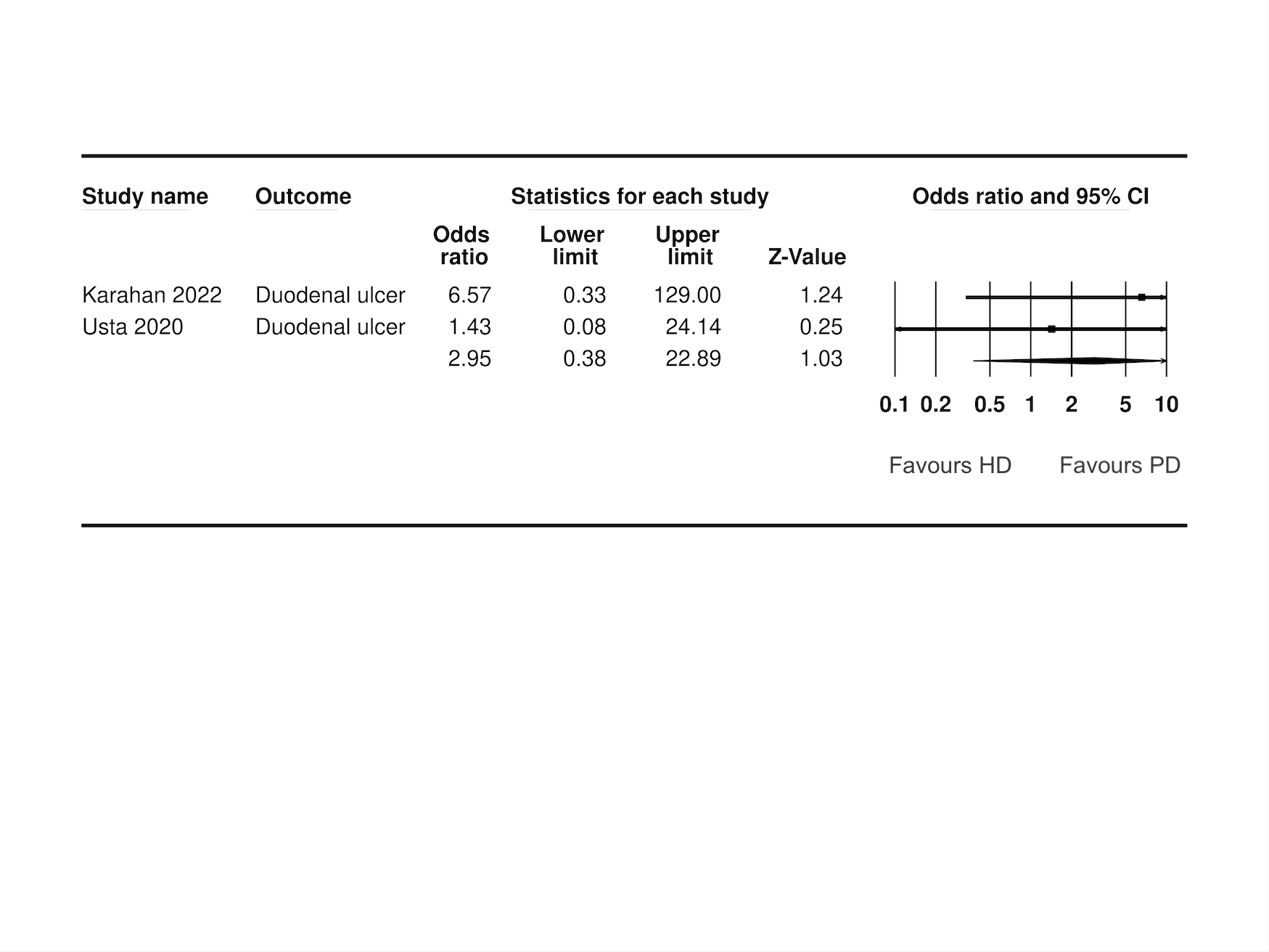

Supplement: Supplemental Information 15 [file peerj-14-21090-s015.tiff]
